# Risk factors for HIV and STI diagnosis in a community-based HIV/STI testing and counselling site for men having sex with men (MSM) in a large German city in 2011–2012

**DOI:** 10.1186/s12879-014-0738-2

**Published:** 2015-01-13

**Authors:** Ulrich Marcus, Jasmin Ort, Marc Grenz, Kai Eckstein, Karin Wirtz, Andreas Wille

**Affiliations:** Department of Infectious Disease Epidemiology, Robert Koch-Institut, P.O. Box 650261, 13302 Berlin, Germany; Berlin School of Public Health, Berlin, Germany; Prävention e.V, Hamburg, Germany; CASA Blanca, Hamburg, Germany; Institut für Hygiene und Umwelt, Hamburg, Germany

**Keywords:** HIV infection, Men who have sex with men-MSM, Sexually transmitted infections, Community based voluntary counselling and testing

## Abstract

**Background:**

In recent years community-based voluntary counselling and testing sites (CB-VCT) for men having sex with men (MSM) have been established in larger cities in Germany to offer more opportunities for HIV testing. Increasingly, CB-VCTs also offer testing for other bacterial sexually transmitted infections. In Hamburg, tests in CB-VCTs are offered free and anonymously. Data on demographics and sexual risk behaviours are collected with a paper questionnaire.

**Methods:**

Questionnaire data from the MSM CB-VCT in Hamburg were linked with serological test results for HIV and syphilis, and with rectal and pharyngeal swab results for gonorrhoea and chlamydia. MSM were defined as males reporting male sex partners. CB-VCT clients were characterized demographically, and associations between sexual behaviour variables and diagnosis of HIV and sexually transmitted infections (STI) were analysed by bivariate and multivariate logistic regression analysis.

**Results:**

Among the male clients of the CB-VCT in 2011–2012 who were tested for HIV or any STI 1476 reported male sex partners. Unprotected anal intercourse (UAI) was reported as reason for testing by 61% of the clients. Forty-one of 1413 clients testing for HIV were tested positive (2.9%). Twenty-four of 1380 clients testing for syphilis required treatment (1.7%). Tests for simultaneous detection of N. gonorrhoea and Chlamydia trachomatis were conducted on 882 pharyngeal and 642 rectal swabs, revealing 58 (=6.6%) pharyngeal and 71 (=11.1%) rectal infections with one or both pathogens. In multivariate logistic regression analysis number of partners, UAI (OR=2.42) and relying on visual impression when selecting sex partners (OR = 2.92) were associated with increased risks for diagnosis of syphilis or a rectal STI. Syphilis or rectal STI diagnosis (OR=4.52) were associated with increased risk for HIV diagnosis.

**Conclusions:**

The MSM CB-VCT in Hamburg reaches clients at high risk for HIV and STIs. The diagnosis of syphilis or a rectal STI was associated with increased odds of testing positive for HIV. Due to the high prevalence of curable bacterial STI among clients and because syphilis and rectal bacterial STI may facilitate HIV transmission, MSM asking for HIV tests in CB-VCTs should also be offered tests for other bacterial STIs.

## Background

In recent years community-based voluntary counselling and testing sites (CB-VCT) [1] for HIV have been established for MSM in larger cities in Germany. One of these CB-VCTs is “Hein&Fiete” in the city of Hamburg. Hein&Fiete was founded in 1990 as an HIV prevention project for MSM. It includes a community drop-in centre with daily opening hours, providing information on gay life in Hamburg, meeting spaces for community groups, outreach prevention work for MSM, HIV and STI serological testing since 2004, and STI swab testing with nucleic acid amplification tests since 2011. The staff consists of approximately 85 volunteers and 5 full or part-time paid staff members. The work of Hein&Fiete, including free testing for HIV and STIs, is funded by the Federal state of Hamburg and by donations.

As low-threshold (anonymous, gay-friendly, low-cost) sexually transmitted infection (STI) screening opportunities for MSM are scarce in the German health care system [2], STI tests, particularly serological tests for syphilis and nucleic acid amplification tests for gonorrhoea and chlamydia infections, are increasingly offered by CB-VCTs. Since these tests can be provided free of charge, uptake of STI tests at Hein&Fiete is the highest among German CB-VCT. To facilitate the pre-test counselling session, clients of CB-VCT sites are invited to fill out an anonymous paper-based and standardized questionnaire on demographics, testing history, transmission risks, and risk management strategies. We analysed these data to identify current risk factors for a diagnosis of HIV and/or STI.

## Methods

### Study procedures

Tests for HIV, HBV, HCV, syphilis, gonorrhoea and Chlamydia trachomatis were offered anonymously and free of charge. Decisions on which tests to take were based on pre-test needs counselling and the clients’ own choice. “Hein&Fiete” provides only testing and counselling and needs to refer clients with an infection diagnosis for further treatment to a private practice or hospital. Because most urethral infections with gonorrhoea and chlamydia in men are expected to be symptomatic, testing for urethral infections with these pathogens is not prioritized, while tests for the mostly asymptomatic pharyngeal and rectal infections are offered. Counsellors recommended pharyngeal and rectal swabs particularly to clients who reported higher numbers of sex partners.

Clients were invited to receive their test results two to five days after testing. In case of a positive test result, adequate therapy options were provided through referral to specialized MSM-friendly physicians.

Questionnaires and test results were entered in a Microsoft Access database by Hein&Fiete staff.

Data were fully anonymized and the anonymous code used at the CB-VCT was exchanged with another unrelated code. Therefore, ethical approval to conduct this analysis was waived.

### Study sample

Study population were all MSM clients attending the CB-VCT of “Hein&Fiete” who were tested for at least one STI (Chlamydia trachomatis, gonorrhoea, HIV, syphilis) and who completed the standardized questionnaire in 2011 and 2012. Men were defined as MSM if they reported at least one male sex partner in the previous 12 months.

### Measurements

Using a 25 item questionnaire, Hein&Fiete collected data on demographics (age, migration status, education), sexual orientation and partnerships, number and gender of sex partners in the previous 12 months, place where sex partners were met, use of drugs in combination with sex, testing history and test results for HIV and STIs, reasons for testing, condom use and reasons for not using condoms, and risk management strategies other than condom use.

Clients were tested for HIV with the Abbott Architect HIV Ag/Ab Combo assay, reactive samples were confirmed by Mikrogen, recomLine HIV-1 & HIV-2 IgG testing. For syphilis testing, samples were screened by Abbott Architect Syphilis TP Reagent, and confirmed by a quantitative TPPA test (MAST). Active infection was defined as either detection of anti-treponemal IgM (Mikrogen, recomWell Treponema IgM) or a positive test for Cardiolipin-KBR (Virion). Pharyngeal swabs taken by medical personnel and rectal swabs taken by the clients themselves were tested for gonorrhoea (Gc) and Chlamydia trachomatis (CT) with the BD ProbeTec™ ET System, which uses strand displacement amplification (SDA) for real-time detection of CT/GC.

### Statistical analysis

The data were analysed using SPSS© (Version 20, IBM Corp.). We used descriptive statistics, calculated bivariate correlations for reporting UAI, being diagnosed with a rectal STI or syphilis, or being diagnosed with HIV, with odds ratios, 95% confidence intervals (CI) and p-values, and constructed three models for multivariate logistic regression analyses to identify factors associated with reporting UAI (principal mode of HIV transmission among MSM), diagnosis of a rectal STI or syphilis (both cofactors for acquiring HIV), and factors associated with diagnosis of HIV. Meeting places were used as binary variables (yes/no). Age and partner number were included as categorical variables (<30 years, 30–44, >44; 0–2 partners, 3–5, 6–10, >10) in all three models (for the age grouping see [3]). Additional factors were only included if they had been significant or borderline significant (p < 0.10) in bivariate analysis. The method used was a conditional forward inclusion (p < 0.05) and backward elimination (p > 0.10) procedure. Reasons for not using condoms during a recent risk situation and risk management strategies other than condom use were not included in the multivariate model for UAI, only in the models for STI and HIV diagnosis.

## Results

A total of 1630 persons filled in a questionnaire at Hein&Fiete in 2011 or 2012. Of these, 1565 men reported on the gender of their sexual partners. There were 1506 men identifiable as MSM. Since not all men filling out the questionnaire were actually tested - some clients were only counselled and some were recommended to get tested later because reported risks were too recent - the final sample for this analysis consisted of 1476 men reporting male partners and having been tested for at least one infection. HIV was tested for most frequently (1413), followed by syphilis (1380), pharyngeal (882) and rectal (642) infections with gonorrhoea and chlamydia. According to self-reported previous test date and place, up to 295 men were tested at least twice at Hein&Fiete during the two years. Numbers and proportions testing positive for the respective infections are presented in Table 1.

**Table 1:** **HIV and STI test results of MSM clients, 2011-2012**

	**Number of tests (any test N = 1476)#**	**Number (%) testing positive**	**Number tested at least twice in 2011-2012**	**Number (%) seroconverting/newly infected**
HIV	1413	41 (2.9)	486*	16 (3.3)
Syphilis (active)	1380	27 (2.0)	271°	3 (1.1)
Syphilis (antibodies)	1380	131 (9.5)	271	n.a.
Gonorrhoea rectal	642	23 (3.6)	154°	8 (5.2)
Gonorrhoea pharyngeal	882	48 (5.4)	212°	16 (7.5)
Chlamydia rectal	642	56 (8.7)	154°	14 (9.1)
Chlamydia pharyngeal	882	15 (1.7)	212°	5 (2.4)
Any rectal STI	642	71 (11.1)		
Any rectal STI or active syphilis	1403	95 (6.8)		

# discrepancies of numbers compared with Figure 1 due to missing age data for 40 clients.

n.a. =not applicable, because treponemal antibodies often persist after treatment. Among the clients reporting testing at least twice 28 (10.3%) had detectable treponemal antibodies. Since the result of the previous tests is unknown, new or re-infection rates could not be determined.

*= 295 had been tested at Hein&Fiete, 191 had been tested elsewhere first and the second time at Hein&Fiete.

°= clients had been tested at Hein&Fiete at least twice in 2011–2012, however it is unknown whether they had been tested twice for this STI or at this location.

The clients having been tested more than once during the two-year observation period may impact the number and proportion of clients testing positive for treponemal antibodies. Based on antibody detection, the new or re-infection rate for syphilis between the repeated tests cannot be determined, and the same individuals may be counted twice or more.

The completeness of core variables in the MSM sample ranged from 82% (response to question on previous chlamydia testing) to 99.9% (sexual orientation). Information e.g. on risk behaviour (unprotected intercourse) and HIV test results were available for approximately 1300 clients, information on rectal STI and risk behaviour for approximately 600 clients.

The median age of the respondents was 36 years (interquartile range: 28–45 years). A migration background (parents or respondent himself immigrated to Germany) was reported by 25% of the respondents. A high school diploma or a higher degree of education was held by 69%. Twelve percent of the sample self-identified as bisexual, 87% as gay. Relationship status at the time of testing was reported as single by 53% of the respondents; another 22% reported being in an open and 25% reported being in a mutually exclusive relationship. A previous test for HIV was reported by 87% of the tested MSM, a previous syphilis test by 59%, 37% had been tested for gonorrhoea, and 31% for Chlamydia trachomatis (both not differentiated by location).

Overall, MSM clients reported a median of 6 and a mean of 11 sexual partners (standard deviation 18) in the previous twelve months. The pharyngeal swab subsample reported a median of 7 and a mean of 13 partners (standard deviation 21), the rectal swab subsample a median of 8 and a mean of 14 partners (standard deviation 24), reflecting the preferential recommendation to take swabs for clients with higher partner numbers.

Transmission risk behaviour, particularly in terms of HIV risks, was queried by a question asking for the reasons for testing. Unprotected anal intercourse (UAI) was reported by 61% of the clients as a reason for testing; 44% reported insertive UAI, and 35% reported receptive UAI. Unprotected oral intercourse with ejaculation into the mouth was reported as risk factor by 33% of MSM. Of note, a large proportion of clients who were diagnosed with a rectal STI did not report receptive UAI (27 out of 71 = 38%). The context in which risks were taken could be derived from questions on drug and alcohol use before or during sex (completed by 65%) and from a question on where most sex partners were met. Bivariate associations of these factors with UAI, STI and HIV diagnosis are shown in Table 2.

**Table 2:** **Results of Bi- und Multivariate analysis of associations between age, partner numbers, meeting places with sex partners, substance use during sex, biological cofactors and risk behaviours with the outcomes UAI, rectal STI and HIV diagnosis**

	**Bivariate**		**Multivariate**		**Bivariate**		**Multivariate**		**Bivariate**		**Multivariate**	
	**UAI**				**STI rectal or syphilis**				**HIV**			
	**OR**	**95% CI**	**OR**	**95% CI**	**OR**	**95% CI**	**OR**	**95% CI**	**OR**	**95% CI**	**OR**	**95% CI**
**Age group**												
<30	1.54***	1.18-1.96	1.67***	1.26-2.23	1.78**	1.12-2.86			1.06	0.49-2.33		
30-44 (ref.)	1				1				1			
>44 years	1.11	0.86-1.43			0.87	0.49-1.54			1.27	0.58-2.80		
**Partner number**												
0-2 (ref.)	1				1				1			
3-5	1.15	0.80-1.64			2.71	0.76-9.66			1.41	0.48-4.14		
6-10	1.18	0.83-1.70			6.10***	1.82-20.41	6.13***	1.79-20.9	0.82	0.25-2.72		
>10	1.59***	1.12-2.25			5.06***	1.51-16.91	5.27***	1.55-18.0	0.91	0.30-2.83		
**Meeting sex partners**												
At friends	1.20	0.93-1.47			1.31	0.85-2.02			0.47*	0.21-1.06		
In bars	1.02	0.81-1.28			0.95	0.60-1.49			0.84	0.42-1.69		
In gay bathhouses	1.05	0.83-1.32			1.25	0.80-1.95			1.08	0.55-2.15		
In porn cinemas	1.07	0.81-1.42			1.11	0.65-1.89			1.83*	0.90-3.71		
In outside cruising sites	1.02	0.71-1.47			0.66	0.28-1.53			1.10	0.39-3.13		
On highway resting areas	1.29	0.67-2.46			1.51	0.53-4.33			0.90	0.12-6.74		
Online	1.49***	1.20-1.84	1.29**	1.01-1.65	1.45*	0.93-2.26			0.81	0.44-1.52		
At parties or discotheques	1.41***	1.12-1.78			0.94	0.60-1.48			0.61	0.29-1.29		
In public urinals	1.55	0.73-3.26			1.34	0.40-4.48			0.97	0.96-0.98		
In fitness centres	1.12	0.75-1.69			0.68	0.27-1.71			0.31	0.04-2.26		
**Substance use**												
Alcohol	1.32***	1.07-1.62			0.82	0.54-1.24			0.63	0.34-1.17		
Inhaled Amyl nitrite	1.68***	1.31-2.15	1.63***	1.23-2.17	1.04	0.65-1.67			1.67	0.88-3.19		
Sildenafil	1.53*	0.96-2.43			1.22	0.55-2.72			1.20	0.36-3.95		
Cocaine, Speed	1.65*	0.93-2.91			2.36**	1.09-5.15			1.21	0.28-5.12		
Cannabis	1.50*	0.97-2.30			1.23	0.58-2.62			1.04	0.32-3.42		
Ecstasy	3.54	0.78-16.03			4.23*	1.15-15.65	5.77**	1.40-23.8	2.83	0.36-22.32		
GBL,GHB	0.61**	0.58-0.63			4.67*	0.93-23.44			4.88	0.59-40.56		
**Cofactors, behaviours**												
Unprotected anal intercourse	n.a.				2.39***	1.46-3.93	2.42***	1.29-4.53	4.52***	1.76-11.59	3.24* °	
STI co-diagnosis	n.a.				n.a.				3.02**	1.22-7.46	4.52***	1.68-12.1
Having sex with partners who appear healthy	n.a.				3.09***	1.94-4.92	2.92***	1.68-5.07	1.27	0.56-2.91		
Asking the insertive partner not to ejaculate inside	n.a.				2.11**	1.16-3.86			4.34***	2.11-8.89	4.44***	1.75-11.3
Asking the partner about HIV status	n.a.				1.71**	1.03-2.85			2.03*	1.00-4.11		
Partner reluctant to use condoms	n.a.				1.30	0.72-2.34			2.68**	1.32-5.45		
No condom available	n.a.				2.81**	1.46-5.41			3.10**	1.26-7.60		

*p > 0.05 < =0.10; **p < =0.05 > 0.01; ***p < =0.01.

n.a. = not applicable.

°Results of Multivariate logistic regression only reported if p < =0.05 (except for UAI as risk factor for HIV diagnosis, p = 0.07).

**Figure 1:**
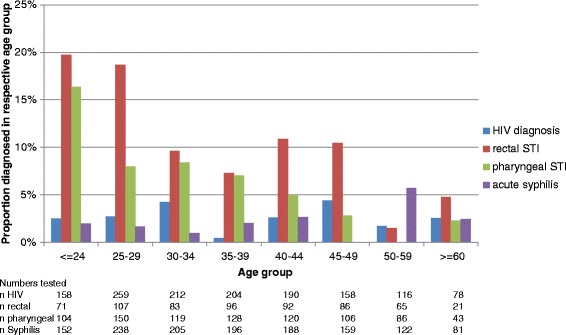
**Proportion of MSM testing positive for different STIs and HIV, by age groups.**

Acute syphilis and HIV diagnoses were almost equally distributed across all age groups in the tested sample. MSM diagnosed with HIV were younger than MSM diagnosed with acute syphilis. Noticeably, while within age groups those men who were diagnosed with rectal or pharyngeal gonorrhoea or chlamydia reported higher numbers of partners, though the differences were statistically not significant, across the whole sample the median number of partners increased with age (from a median of 5 for age <30 years to a median of 10 for age 45–59), while the proportion of clients diagnosed with gonorrhoea and chlamydia decreased (Figure 1). Please note that numbers do not sum up to the numbers in Table 1 due to missing age data of 40 clients.

Clients reporting a self-perceived risk situation as a reason for testing (compared to routine testing or testing when entering a new partnership) were significantly more likely to be diagnosed with HIV and STI (OR = 2.8; 95% CI 1.4-5.6). In bivariate analysis, reported reasons positively associated with risk situations were reluctance of the sex partner to use a condom and unavailability of condoms at a sexual encounter (see Table 2).

Among the risk management strategies besides condom use, asking the active partner to withdraw before ejaculation and asking the partner about his HIV status were positively associated with being diagnosed with a bacterial STI or with HIV (Table 2).

Reported UAI was strongly associated with an HIV diagnosis and less strongly associated with the diagnosis of a rectal STI or syphilis. The concomitant diagnosis of a rectal infection with gonorrhoea or chlamydia or a diagnosis of syphilis was strongly associated with an HIV diagnosis. For clients reporting an HIV positive partner the odds ratio for being diagnosed with HIV was 1.8 (95% CI 0.8-4.1; p = 0.175) compared to others, which did not reach statistical significance.

In multivariate logistic regression analyses including age and partner numbers (significantly associated with UAI and STI diagnoses in bivariate analysis) in addition to reported substance use and risk management strategies (the latter not included for UAI, but only for STI and HIV diagnosis outcomes) showing statistical significance in bivariate analysis, the following factors remained significantly associated (results see also Table 2)

with UAI: age group, a high partner number, and use of inhaled amyl nitrite (poppers);

with diagnosis of a rectal STI or syphilis: high partner numbers, reporting use of ecstasy when having sex, reporting UAI, and having sex only with partners who appear healthy;

with HIV diagnosis: diagnosis of rectal gonorrhoea or chlamydia infection or diagnosis of syphilis; asking the active partner to withdraw before ejaculation. The odds ratio for being diagnosed with HIV when reporting UAI was 2.8, but this did not reach statistical significance (95% CI 0.8-10.1).

## Discussion

The data show that the CB-VCT site in Hamburg reached MSM at high risk for HIV and bacterial STI. Though a cost-effectiveness analysis was not conducted, considering the high prevalence and low overhead costs of the CB-VCT site, testing at the CB-VCT is likely a very cost-effective way of reducing the proportion of undiagnosed HIV and STI infections in this population compared to untargeted screening in medical facilities and also compared to untargeted VCT sites [4-9]. However, well-educated and self-identified gay men were reached disproportionally: a high school diploma or a higher degree of education was held by 69%, compared to 35% in the German general population and 60% in a recent MSM online survey (“Gay men and AIDS 2013”- GMA 2013, unpublished observations). Eighty seven percent self-identified as gay, while large population-based surveys suggest a proportion of non-gay-identified MSM which is clearly higher than 13% [10]. Thus, to reach a broader spectrum of MSM, the current CB-VCT approach may need to be combined with other approaches to reach MSM who are less well educated and less out about their sexual preferences than the clients reached by Hein&Fiete.

Many CB-VCT sites in Europe so far only offer HIV testing, sometimes in combination with syphilis screening. The Hamburg experience, finding higher prevalence for rectal and pharyngeal infections with gonorrhoea or chlamydia than for syphilis or HIV (though in a more preselected subsample) convincingly argues for the combination of HIV and STI testing for MSM. Our data show that a more comprehensive offer to screen for all relevant bacterial STIs is warranted for this population. While testing costs have not been an issue so far in Hamburg where tests are covered by public funds, nucleic acid amplification tests for gonorrhoea and chlamydia may increase testing costs considerably, particularly when all three possibly affected sites (urethra, pharynx, and rectum) are screened. One option to minimize costs might be the use of pooled combination tests, i.e. pooling pharyngeal, rectal and urethral swabs or a urine specimen for analysis and using combination tests for gonorrhoea and chlamydia [11]. Selective offering of tests according to reported sexual risks could be another option. However, a large proportion of clients who were diagnosed with a rectal STI did not report receptive UAI. Reasons for this may be responses biased by social desirability to underreport UAI in general as well as a lack of questions that would cover all possible modes of transmission of bacterial STI to the rectal mucosa beyond insertive UAI.

We found an interesting inverse association between median numbers of partners and the proportion of rectal and pharyngeal infections with gonorrhoea and chlamydia by age group: although MSM from older age groups reported higher median numbers of partners, they were less likely to be diagnosed with rectal or pharyngeal infections. Similar observations have already been reported by other authors [12,13]. However, within the same age groups MSM who were diagnosed with rectal or pharyngeal infections reported higher median numbers of partner than MSM who were not diagnosed with an infection. This is reminiscent of the age-dependence of chlamydia diagnoses in women, which might be explained by an evolving immunity to this pathogen by repeated exposures over time [14]. Other reasons for this inverse association could be differences in condom use or partner selection for anal intercourse without a condom by age group. However, lack of condom use would not explain the differences for pharyngeal infections, since condoms are very rarely used for oral intercourse regardless of age group.

The associations we found between risk management strategies aiming at identifying partners infected and diagnosed with HIV and outcomes such as UAI, rectal STI or HIV diagnosis may reflect how widespread these risk management strategies are, that they are ineffective for avoiding other STI, and that even their utility to avoid HIV infection may be very limited. A high proportion of MSM diagnosed with HIV receives antiretroviral treatment and may thus be only minimally infectious, while most men who are still infectious are not yet diagnosed and thus not identifiable by respective questions. Our finding of a non-significant association of testing HIV positive when a partner is known to be infected with HIV is in contrast to a very high hazard ratio for testing HIV positive when reporting unprotected anal intercourse with a known HIV positive partner in the United States-based EXPLORE study conducted from 1999 through 2003 [15]. The main explanation for this discrepant finding may be the increased treatment rate among MSM in Germany compared to the situation in the United States ten years ago.

Contrastingly, men who have multiple partners and rely predominantly on HIV serosorting instead of condom use for protection against HIV transmission may be more likely to meet partners using the same risk management strategy. Once HIV is present in sexual networks using this risk management strategy, it may spread quite efficiently, particularly because other bacterial STIs accumulating in these networks are further enhancing HIV transmission. Alternatively or additionally, rectal STIs may just be a marker for risk behaviours or networks with increased HIV transmission risks. Other STIs, particularly rectal STIs, have been reported to be associated with an increased risk of acquiring HIV, e.g. by a group from New York in a longitudinal study design [16].

The association of STI diagnosis (rectal STI or syphilis) with the risk management strategy ‘having sex only with partners who appear healthy’ in multivariate regression analysis is a reminder that infections with bacterial STI are even less visible than HIV infection. Relying on visual appearance is therefore particularly ineffective for avoiding STI infections. The strong association between HIV diagnosis and the risk management strategy of asking the active partner to withdraw before ejaculation is difficult to interpret: the format of the question leaves open whether this strategy refers to oral or anal intercourse or both. Since a causal association with HIV transmission is conceivable only for anal intercourse, this association might be confounded. Asking for withdrawal could just be a surrogate for other unmeasured characteristics or risk management strategies of men at increased risk for HIV.

Another interesting finding is the subtle discrepancy between meeting places as risk factors for STI and for HIV diagnosis in the bivariate analysis, considering only the results which reached borderline statistical significance. While discrepancies in statistical significance regarding risk factors may just be due to the lower numbers of HIV compared to STI diagnoses, it could also be an indication of subtle differences regarding HIV and STI transmission risks in different settings. High STI transmission risks would be expected in settings with low condom use due to high probability of HIV serostatus communication (e.g. when meeting partners online and in private settings [17]. High HIV transmission risk was associated with meeting partners at porn cinemas, which in Hamburg is probably the most popular sex-focused gay venue with only minimal serostatus communication.

There are of course several limitations to this investigation: the questionnaire was primarily designed to facilitate clients’ sexual-health counselling needs, not to systematically detect specific risk factors. Associations we detected, particularly between risk management strategies and STI and HIV diagnosis, may therefore be confounded by other, unmeasured factors. It is likely that some questions were answered with regard to recent situations where respondents assumed risks; however this does not necessarily mean that HIV or STIs were transmitted during these encounters. The use of a combined outcome (rectal STI or syphilis), in which the number of men tested for rectal STI was less than half of the number of men tested for syphilis weakens associations, because many rectal STIs may have remained undiagnosed. Using only rectal STI as outcome changes some of the associations in bivariate analysis, but has no substantial impact on the variables remaining significant in multivariate analysis (data not shown). A social desirability bias, particularly regarding the reporting of unsafe sexual practices and drug use, should be expected, since the responses were known to be discussed with a sexual-health counsellor. That UAI was not significantly associated with HIV diagnosis in the multivariate regression model may be an indication of UAI underreporting. Lastly, with its focus on common sexual practices among MSM, the scope of sexual practices that could possibly result or contribute to the transmission of bacteria to the rectum or pharynx was not fully explored [18].

## Conclusions

Our results emphasize the need to combine HIV and STI testing for MSM and the need for comprehensive screening for mostly asymptomatic rectal and pharyngeal infections [19]. Community-based VCTs can reach the MSM target group quite efficiently, and offering comprehensive HIV and STI testing in this setting is likely to be much more cost effective than relying on traditional health care settings. However, the CB-VCT approach for MSM is likely only feasible in larger cities with respective gay communities. Additional alternative approaches will be necessary to reach rural MSM, MSM less connected to the gay community, and MSM less willing to self-identify as gay. Behavioural data which may be useful to adapt and fine-tune prevention messages for MSM can also be collected in this setting. The data collected routinely before the counselling session appears quite useful for the analysis of risk behaviours and trends in risk management strategies, especially combined with testing data.
